# Ligand Modulated Antagonism of PPARγ by Genomic and Non-Genomic Actions of PPARδ

**DOI:** 10.1371/journal.pone.0007046

**Published:** 2009-09-16

**Authors:** Mattias C. U. Gustafsson, Deborah Knight, Colin N. A. Palmer

**Affiliations:** Biomedical Research Institute, University of Dundee, Ninewells Hospital and Medical School, Dundee, United Kingdom; Centre National de la Recherche Scientifique, France

## Abstract

**Background:**

Members of the Peroxisome Proliferator Activated Receptor, PPAR, subfamily of nuclear receptors display complex opposing and overlapping functions and a wide range of pharmacological and molecular genetic tools have been used to dissect their specific functions. Non-agonist bound PPARδ has been shown to repress PPAR Response Element, PPRE, signalling and several lines of evidence point to the importance of PPARδ repressive actions in both cardiovascular and cancer biology.

**Methodology/Principal Findings:**

In this report we have employed transient transfections and luciferase reporter gene technology to study the repressing effects of PPARδ and two derivatives thereof. We demonstrate for the first time that the classical dominant negative deletion of the Activation Function 2, AF2, domain of PPARδ show enhanced repression of PPRE signalling in the presence of a PPARδ agonist. We propose that the mechanism for the phenomenon is increased RXR heterodimerisation and DNA binding upon ligand binding concomitant with transcriptional co-repressor binding. We also demonstrated ligand-dependent dominant negative action of a DNA non-binding derivative of PPARδ on PPARγ1 signalling. This activity was abolished upon over-expression of RXRα suggesting a role for PPAR/cofactor competition in the absence of DNA binding.

**Conclusions/Significance:**

These findings are important in understanding the wide spectrum of molecular interactions in which PPARδ and PPARγ have opposing biological roles and suggest novel paradigms for the design of different functional classes of nuclear receptor antagonist drugs.

## Introduction

The peroxisome proliferator-activated receptors (PPARs) α, δ and γ belong to the nuclear receptor family of transcriptional regulators. They function as obligate heterodimers with the retinoid X receptors, RXRs, and signal from PPAR response elements (PPREs) upon binding PPAR- and/or RXR agonists. The PPAR ligands consist of naturally occurring fatty acids and fatty acid derivatives as well as a range of synthetic drugs [1], [2], [3].

PPARα is involved in the control of catabolic fatty acid metabolism such as peroxisomal β-oxidation and mitochondrial β-and ω-oxidation of fatty acids and is most prevalent in metabolically active tissues such as liver. PPARα is activated by the blood lipid lowering fibrate drugs. These acts as peroxisome proliferators in mice and rats but no adverse effects have been detected in human livers [1], [4].

PPARγ is involved in fatty acid and glucose homeostasis and is required for adipocyte differentiation and for placental development. Activation of PPARγ also seems to act anti-inflammatory and to hinder proliferation or cause apoptosis in cancer cells. The insulin sensitizing thiazolidinedione drugs, which are high affinity PPARγ agonists, are used to treat type 2 diabetes and experimentally to treat cancer [5].

PPARδ is widely expressed and the most prevalent PPAR in several tissues both in the adult organism and during development [6]. It is also the least known in terms of biological function, although recent reports would suggest that it might have a role similar to PPARα in tissues other than liver. PPARδ has also been shown to be involved in placental implantation, wound healing, and carcinogenesis [4], [7], [8], [9]. No PPARδ ligands are currently used as such in treatment of disease, although studies on human subjects for the use of a PPARδ agonist in the treatment of metabolic syndrome have been reported [10], [11].

Recently, it was shown that non-liganded PPARδ attracts transcriptional co-repressors when bound to DNA more effectively than PPARα and γ. Due to its widespread distribution it was suggested that PPARδ acts as a PPRE gateway receptor [12], [13]. Given the, sometimes conflicting, results on PPARδ biology obtained using various pharmacological and molecular genetic tools we set out to study the ligand modulated antagonism of PPARγ1 by genomic and non-genomic actions of PPARδ. We found in accordance with [13] that non-liganded PPARδ represses PPARα and γ. In line with this the PPARδ derivative PPARδΔAF2, lacking helix 12 (or activation function 2, AF2), acts dominant negatively on PPARα, γ1 and δ signalling. Furthermore, we found that PPARδΔAF2 possess ligand enhanced dominant negative activity on PPRE signalling. In contrast to Shi et al. [13] who reported that a non-DNA binding PPARδ derivative didn't exert any dominant negative effects, we found that non-DNA bound PPARδ ligand-binding domain (LBD) exerts ligand-dependent dominant negative activity on PPARγ1 signalling. Since PPARδ and γ co-exist in a range of tissues and in many cases have opposite biological effects we propose that the phenomena discovered might have important implications for PPAR experimental designs, PPAR biology in general and possibly drug design.

## Results and Discussion

### Agonist non-bound PPARδ is a repressor of PPARγ1 dependent PPRE signalling, but not vice versa

**Figure 1:**
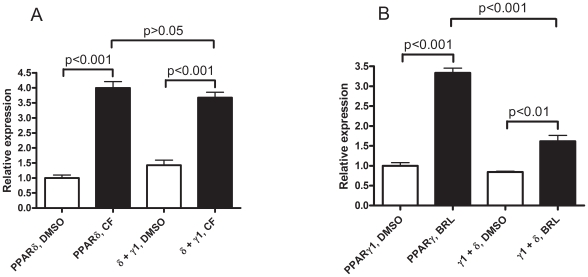
The effect of (A) non-liganded PPARγ1 on PPARδ signalling and of (B) non-liganded PPARδ on PPARγ1 signalling. COS-1 cells were transiently transfected with (per well in six-well plates) 50 ng (A) pCLDN-hPPARδ or (B) pCDLN-hPPARγ1 and 250 ng (A) pCLDN or pCLDN-hPPARγ1 and (B) pCLDN or pCLDN-hPPARδ, respectively.

Due to its widespread tissue distribution and the fact that it interacts more efficiently on DNA with nuclear receptor co-repressors than the other PPAR isoforms it was proposed, as well as demonstrated *in vitro*, that PPARδ functions as a PPRE gateway receptor [12], [13]. We confirmed this phenomenon for PPARδ and γ1 signalling using transient transfection of COS-1 cells with plasmids encoding these PPAR isoforms and a promiscuous (transcriptionally transactivated by all three PPAR isoforms, data not shown for PPARα), PPRE luciferase reporter gene construct (pLFABPluc). We found that the presence of unliganded PPARγ1 did not affect PPARδ signalling (Figure 1A) whereas unliganded PPARδ significantly (P<0.001) repressed the PPARγ1 dependent signalling from pLFABPluc (Figure 1B).

### Ligand-enhanced dominant negative action of PPARδΔAF2

**Figure 2:**
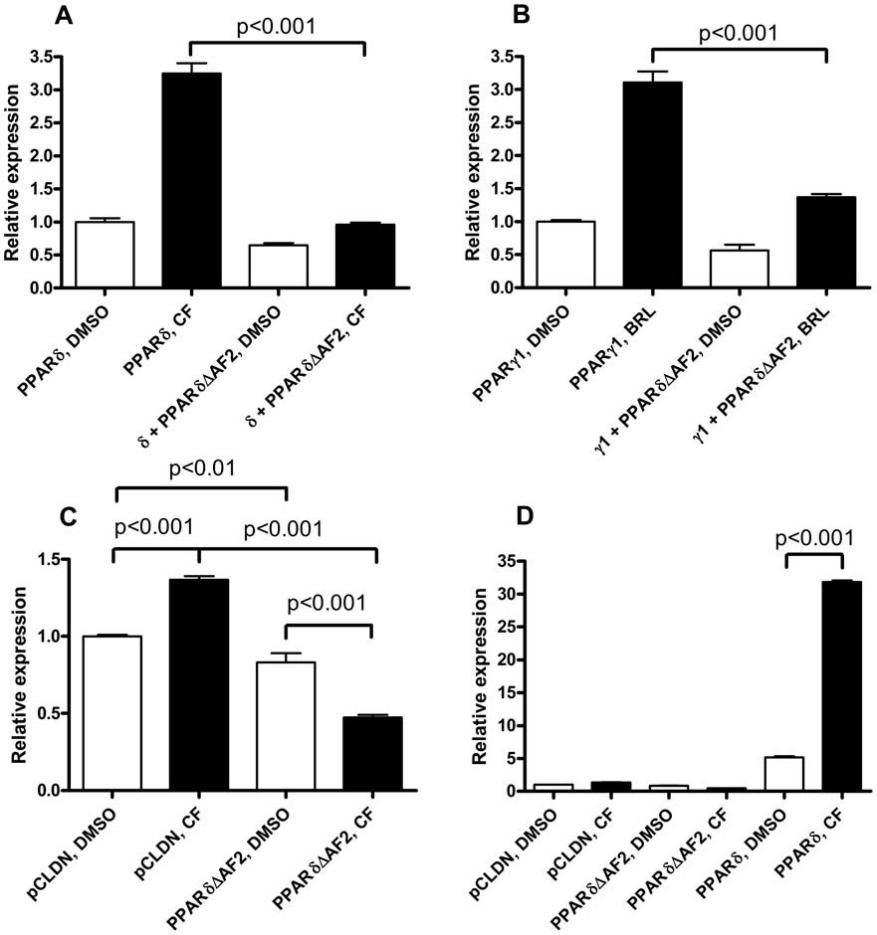
PPARδΔAF2 represses (A) PPARδ and (B) PPARγ1 signalling. (C) PPARδΔAF2 represses TK-promoter activity in a ligand-enhanced fashion. COS-1 cells were transiently transfected with (per well in six-well plates) (A) 50 ng pCLDN-hPPARδ or (B) pCDLN-hPPARγ1 and 250 ng pCLDN or pCLDN-hPPARδΔAF2. (C) and (D) T47D cells were transfected with (per well in a six-well plate) 500 ng pCLDN, pCLDN-hPPARδΔAF2 or pCLDN-hPPARδ. (D) is identical to (C) except for the two additional bars representing over-expression of PPARδ with and without CF.

Helix 12 modifications (both designed and for PPARγ, found in human patients as mutations) have been shown to render PPARs dominant negative due to their inability to recruit co-activators while retaining the ability to bind co-repressors [14], [15], [16]. Given the superior repressing properties of PPARδ, modification of helix 12 should render it a relatively effective ligand independent repressor of PPRE signalling. We have previously employed a PPARδ derivative lacking the C-terminal 11 amino acid residues, PPARδΔAF2, as a tool for studying PPRE signalling [17]. In order to further characterize the properties of this construct we conducted a range of transient transfection experiments. PPARδΔAF2 was found to act in a dominant negative fashion on PPARα, γ1 and δ signalling (Figure 2A & B, respectively, P<0.001, data not shown for PPARα), thus confirming and extending our previous observations.

Upon agonist binding PPARs undergo a conformational change leading to increased RXR heterodimerisation and shedding of transcriptional co-repressors with the subsequent recruitment of transcriptional co-activators [3]. The increased PPAR-RXR heterodimerisation leads to an increased affinity for PPREs [18], [19]. This would in the case of PPARδΔAF2 lead to increased occupancy of the PPREs concomitant with recruitment of transcriptional co-repressors and thus further reduced PPRE signalling. We thus investigated the effect of a PPARδ agonist on the dominant negative properties of PPARδΔAF2. Because of the relatively high endogenous PPRE signalling in the COS-1 cells we employed T47D cells grown in RPMI 1640 medium supplemented with 5% dextran charcoal-stripped serum for this experiment. The effect of over-expressing and transactivating PPARδ in T47D cells is shown in Figure 2D. We could detect a small but significant (P<0.001) PPARδ (CF dependent) activity in cells with no added PPARδ expression vector (Figure 2C). We could also see a small but significant (P<0.01) effect of introducing PPARδΔAF2 on non-CF dependent transcription of the luciferase gene in pLFABPluc (Figure 2C). The dominant negative effect of introducing PPARδΔAF2 into the system was further enhanced by the addition of CF (P<0.001). This indicates that for PPARδΔAF2 CF acts as an inverse agonist that enhances the dominant negative effect, a novel concept for type II nuclear receptors. The concept was discussed and investigated for the only PPARδ antagonist described to date, GSK0660. GSK0660 did not, however, increase occupancy of PPARδ or transcriptional co-repressors to chromatin PPREs [20].

### The PPARδ ligand-binding domain is a repressor of PPRE dependent PPARγ1 signalling in the presence of a PPARδ agonist

**Figure 3:**
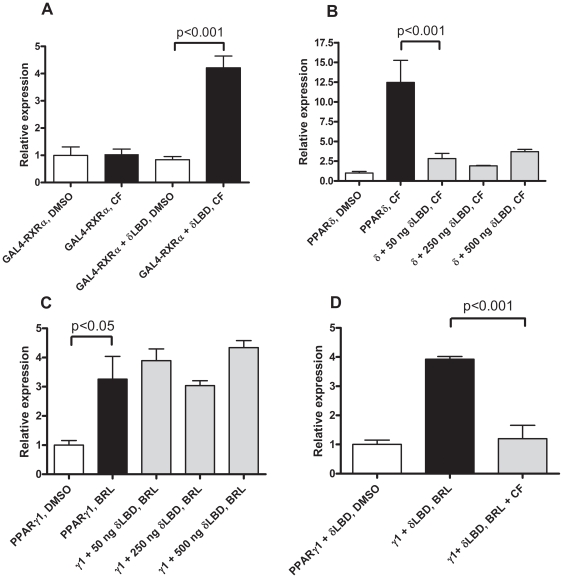
(A) The PPARδ LBD is functional with respect to transcriptional transactivation and RXR heterodimerisation and (B), (C) and (D) possess ligand-dependent dominant negative behaviour. (A) COS-1 cells were transfected with 500 ng pCMVgRXR and 500 ng pCLDN or pCLDN-δLBD. (B), (C) and (D) COS-1 cells were transiently transfected with: (B) 500 ng pJ3Nuc (hPPARδ expression plasmid) and 0 to 500 ng pCLDN or pCLDN-δLBD; (C) 50 ng pCLDN-hPPARγ1 and 0 to 500 ng pCLDN or pCLDN-δLBD; (D) 50 ng pCLDN-hPPARγ1 and 500 ng pCLDN-δLBD.

Since the PPARs act as RXR heterodimers it would be conceivable that RXR competition could occur among the PPAR isoforms. In fact, ligand dependent RXR competition has been described for PPARα and liver X receptor (LXR) [21], [22], PPARβ/δ and LXRα [23], PPARα and thyroid hormone receptor (TR) [24] as well as PPARγ and TRα1 and β mutants [25], [26]. Agonist-bound wild-type PPARδ and γ activate transcription when bound to PPREs. Thus, in order to study the PPRE independent effects of PPARδ and γ we needed a non-DNA binding derivative with a functional ligand binding and activating domain. We generated an expression plasmid for the PPARδ LBD, pCLDN-δLBD, and tested it for the desired properties in a mammalian two-hybrid assay. Co-expression of the GAL4-RXRα fusion protein and the PPARδ LBD led to CF induced upstream activating sequence (UAS) dependent transcriptional transactivation, strongly indicating that the PPARδ LBD is functional with respect to RXR heterodimerisation and transcriptional co-activator recruitment (Figure 3A, P<0.001).

Subsequent to the functional validation of the PPARδ LBD we investigated whether it had a dominant negative effect on PPARδ and γ1 signalling. We found that PPARδ but not PPARγ1 signalling was abolished by co-expression of the PPARδ LBD (Figures 3B (P<0.001) and C, respectively). One important difference between the experiments in Figures 3B and C is the absence of the CF in 3C. If a PPARδ agonist is required for efficient RXR heterodimerisation then the addition of CF would render the PPARδ LBD dominant negative on PPARγ1 signalling. Indeed, we found that the PPARδ LBD could repress the PPARγ1 signalling in the presence of a PPARδ agonist (Figures 3D, P<0.001).

**Figure 4:**
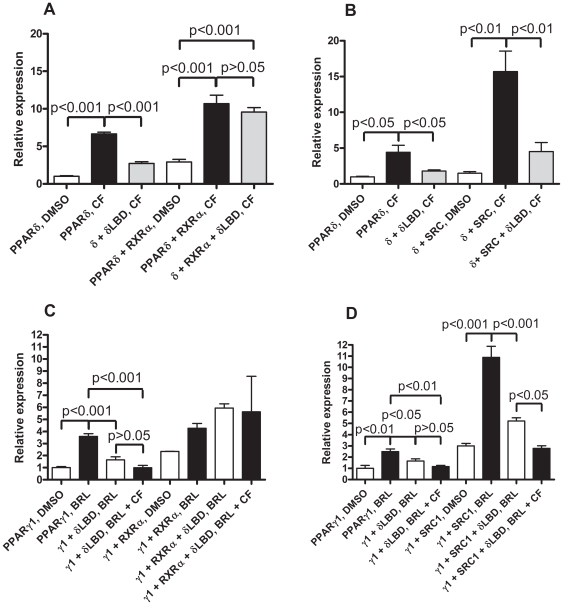
The effect of co-expression of RXRα and SRC1a on PPARδ LBD mediated repression of PPARδ (A and B) and PPARγ1 (C and D) signalling, respectively. COS-1 cells were transfected with: (A and B) 500 ng pJ3Nuc and the following plasmids: 500 ng pCLDN or pCLDN-δLBD and pCLDN or (A) pSG-mRXRα or (B) pSG5-SRC1a and for (C and D) 50 ng pCLDN-hPPARγ1 and the following plasmids: 500 ng pCLDN or pCLDN-δLBD and 500 ng pCLDN or (C) pSG-mRXRα or (D) pSG5-SRC1a.

Given the known effects of agonist binding to a PPAR one could speculate whether the dominant negative effect of the PPARδ LBD is due to RXR or transcriptional co-activator squelching. To address this question we co-expressed RXRα and the transcriptional co-activator, steroid receptor co-activator 1a (SRC1a), with PPARδ and γ1 with and without the PPARδ LBD. PPARδ signalling was found to be repressed by co-expression of the PPARδ LBD (Figure 4A and B, P<0.001 and P<0.05, respectively). This dominant negative effect was abolished by co-expression of RXRα (Figure 4A, P>0.05). Co-expression of SRC1a with PPARδ increased the agonist dependent inducibility of reporter activity but didn't abolish the effects of PPARδ LBD dependent repression (Figure 4B).

We then proceeded to study the effect of RXRα and SRC1a co-expression on the effect of the PPARδ LBD on PPARγ1 signalling. In this experimental setup the PPARδ LBD showed dominant negative behaviour in the absence of CF (Figures 4C and D, P<0.001 and P<0.05, respectively). The dominant negative effect of the PPARδ LBD was somewhat enhanced by the PPARδ agonist (Figures 4C and D). The effect of co-expression of RXRα was similar to that of the PPARδ experiment with overall activity somewhat increased but with lower levels of PPARγ agonist dependent induction and in abolishing the dominant negative effect of the PPARδ LBD (Figure 4C). Co-expression of SRC1a increased the level of activity of PPARγ1 without having a much of an effect on the level of induction (Figure 4D). The PPARδ LBD repressed PPARγ1 signalling (P<0.05) with additional repression seen in the presence of CF (Figure 4D). As was the case for PPARδ, the addition of SRC1a increased the overall levels of signalling (Figure 4D). Also similarly with the SRC1a co-expression experiment with PPARδ the addition of SRC1a did not abolish the PPARδ LBD mediated repression. Instead, the level of PPARδ LBD mediated repression became more pronounced (Figure 4D, P<0.001). Furthermore, the PPARδ agonist enhanced repression was more marked (Figure 4D, P<0.05). Since the addition of RXRα seems to relieve the PPARδ LBD mediated repression of PPARδ and PPARγ1 signalling whereas the addition of SRC1a still allows the PPARδ LBD mediated repression we conclude that RXR sequestration is likely to be the main mechanism behind the phenomenon. We thus speculate that ligand dependent RXR competition could occur *in vivo* between at least PPARδ and PPARγ and quite possible between all three PPAR isoforms.

### Concluding remarks

The major conclusion we draw from this study is that care must be taken when interpreting results obtained from all genetic models of PPARδ action. The genetic ablation of PPARδ will remove both the ability to activate PPARδ, but also the intrinsic role that PPARδ has in the tempering of PPARα and PPARγ signalling. Therefore it is prudent to use a wide range of both gain and loss of function experiments in order to fully understand the function of PPARδ and its relationship to PPARα and PPARγ signalling. This is most likely to be true for other nuclear receptors forming heterodimers with RXRs as well.

Our study also might suggest a novel paradigm for the design of different functional classes of type II nuclear receptor antagonist drugs. One could envisage two sets of nuclear receptor antagonists with very different biological actions (simplistically stating the two extremes of antagonist behaviour); one that displaces the PPAR/RXR complex from the PPRE and one that simultaneously increases DNA binding and transcriptional co-repressor recruitment.

## Materials and Methods

### Cloning and plasmids

General DNA techniques were performed according to [27]. DNA sequencing was done by the DNA Analysis Facility, Human Genetics Unit, at Ninewells Hospital, Dundee. *Escherichia coli* XL1 Blue was transformed according to the manufacturer's instructions (Stratagene).

The expression plasmids pCLDN-hPPARδ (pMGD60), pCLDN-hPPARδΔAF2, pCLDN-hPPARγ1, pJ3NUC, pCMVg-RXR, pSG-mRXRα and pSG5-SRC1a as well as the PPRE reporter plasmid pLFABPluc have been described previously [17], [28], [29], [30], [31], [32], [33]. The internal transfection control plasmid pSVβ-galactosidase is from Promega. The part of human *PPARδ* encoding the LBD (from codon A142, including an added translational start codon, in bold) was amplified with primers PRMG4 (5′-CGGGGTACC **ATG**GCTATCCGTTTTGGTCGGATG-3′) and PRMG5 (5′-CGGGGTACCTTAGTACATGTCCTTGTAGATCTCC-3′) (*Kpn*I-sites underlined). The *Kpn*I cleaved PCR product was cloned into pCLDN [34], creating pCLDN-δLBD (confirmed by sequencing). A GAL4-fusion luciferase reporter plasmid (p4×UAS-TK-luc) was constructed by cloning the *Sal*I-*Xho*I fragment of pLacZr [30] (containing the 4×UAS-TK, Upstream Activating Sequence) module in pGL3basic (Promega) cleaved with *Xho*I.

### Growth of cells and transient transfections

COS-1 and T47D cells (Cancer Research U. K. cell resources unit) were grown in a 5% CO_2_ atmosphere at 37°C in high glucose DMEM supplemented with 10% foetal bovine serum and 50 U/ml penicillin G and 50 µg/ml streptomycin (Gibco) and 2 mM L-glutamine for COS-1 and T47D cells, respectively. For transfections the T47D cells were grown in RPMI 1640 (phenol red-free) containing 5% dextran-charcoal stripped foetal bovine serum. Transient transfections of COS-1 cells and T47D cells were performed in six-well plates using DEAE-dextran according to Cullen [35] and Lipofectamine 2000 (Invitrogen), respectively. 24 hours post transfection, medium containing 50 nM compound F, CF, [33] for PPARδ activation and/or 500 nM rosiglitazone, BRL, [36] for PPARγ1 activation in a final concentration of 0.1% dimethyl sulfoxide (DMSO) or DMSO alone was added. 48 hours post transfection cell lysates were generated using Promega's reporter lysis buffer.

For all transfections 500 ng luciferase reporter (pLFABPluc or p4×UAS-TK-luc) and 50 ng pSVβ-galactosidase were used per well in six-well plates. Luciferase activity was assayed with the Promega luciferase assay substrate and β-galactosidase activity according to Sambrook et al. using o-nitrophenyl-β-D-galactopyranoside [27] or using the chemiluminescent β-gal reporter gene assay kit from Roche.

### Statistical analysis

Relative reporter gene expression is stated as the luciferase activity normalized against the corresponding β-galactosidase activity. These values have in turn been normalised against the mean of the normalized luciferase activities of the leftmost bars in each graph. Each experiment was repeated three times and the bars in the graphs represent the means and the error bars represent the standard error of the mean. One-way ANOVA was performed on the data from each experiment and the Newman-Keuls test was employed for calculating statistical significance using GraphPad Prism 3 software.
